# Breakthroughs and challenges of CAR-T cell therapy in treating hematologic malignancies of the central nervous system

**DOI:** 10.1097/BS9.0000000000000289

**Published:** 2026-04-27

**Authors:** Xiaoning Li, Haili Da, Jie Zhao, Yan Yan, Jia Wei, Weiwei Tian

**Affiliations:** a Third Hospital of Shanxi Medical University, Shanxi Bethune Hospital, Shanxi Academy of Medical Sciences, Tongji Shanxi Hospital, Taiyuan 030032, China; b Fenyang Hospital of Shanxi Province, Fenyang 032200, Shanxi, China; c Shanxi Bethune Hospital, Shanxi Academy of Medical Sciences, Third Hospital of Shanxi Medical University, Tongji Shanxi Hospital, Taiyuan 030032, China; d Tongji Hospital, Tongji Medical College, Huazhong University of Science and Technology, Wuhan 430030, China.

**Keywords:** Blood–brain barrier, Central nervous system, Chimeric antigen receptor T, Leukemia, Lymphoma, Multiple myeloma, Neurotoxicity

## Abstract

Chimeric antigen receptor T (CAR-T) cell therapy has transformed hematologic cancer treatment; however, its application in patients with central nervous system (CNS) involvement remains challenging because of exclusion from key trials. Recent data show that CAR-T cells can breach the blood–brain barrier (BBB) and achieve clinically significant responses in CNS lymphoma, acute lymphoblastic leukemia (ALL), and multiple myeloma (MM). However, limited CNS trafficking, antigen escape, an immunosuppressive microenvironment, and treatment-related neurotoxicity constrain therapeutic efficacy. This review synthesizes recent clinical outcomes, elucidates mechanisms of CNS infiltration and neurotoxicity, and evaluates emerging strategies, including optimized CAR designs, BBB-modulating technologies, alternative delivery routes, and rational combination therapies, to enhance safety and efficacy. Disease-specific nuances, such as Parkinson-like symptoms in CNS myeloma and differential neurotoxicity profiles between primary and secondary CNS lymphomas, are highlighted. By identifying key knowledge gaps and proposing prioritized research directions, this study aims to guide the future translation of CAR-T therapy for CNS hematologic malignancies.

## 1. INTRODUCTION

Chimeric antigen receptor T (CAR-T) cell therapy has progressed from concept to clinical reality, with 11 approved products demonstrating remarkable efficacy against relapsed/refractory B-cell malignancies. However, patients with central nervous system (CNS) involvement were excluded from key trials; therefore, the role of CAR-T remains unclear.

Treating CNS malignancies presents unique challenges, including the blood–brain barrier (BBB), an immunosuppressive microenvironment, and distinctive neurotoxicity risks. However, emerging evidence confirms that CAR-T cells can traverse the BBB and achieve meaningful clinical responses. In relapsed/refractory CNS lymphoma, objective response rates (ORR) reach 64%, with complete remission (CR) rates of 31% to 40%.^1^ In CNS acute lymphoblastic leukemia (ALL), CAR-T therapy extends median overall survival (OS) to 16 months, achieving CNS and bone marrow remission rates of 85.4% and 87.5%, respectively.^2^ In small, early-stage multiple myeloma (MM) cohorts, BCMA-targeted CAR-T therapy has shown high CNS response rates. However, larger studies remain warranted.^3^

Despite these promising outcomes, inadequate CNS trafficking, antigen escape, and toxicities, such as cytokine release syndrome (CRS) and immune effector cell-associated neurotoxicity syndrome (ICANS), constrain therapeutic efficacy. This review synthesizes the rapidly evolving application of CAR-T therapy for CNS hematologic malignancies. We delineate the mechanisms of CNS trafficking and neurotoxicity, appraise disease-specific clinical outcomes, and evaluate translational strategies to overcome key hurdles. This study may guide future research and accelerate the effective clinical translation of these therapies.

## 2. MECHANISMS OF CAR-T THERAPY IN CNS HEMATOLOGIC MALIGNANCIES

CAR-T cell therapy exerts antitumor effects in CNS hematologic malignancies primarily by direct tumor cell killing and remodeling the immune microenvironment.

Before CAR-T cell infusion, the BBB in CNS malignancies is often compromised owing to tumor-associated inflammatory remodeling, primarily driven by tumor cells and activated astrocytes, which upregulate vascular endothelial growth factor (VEGF)-A and other inflammatory mediators, activating vascular endothelial growth factor receptor-2 (VEGFR-2)/phosphatidylinositol 3-kinase (PI3K)/protein kinase B (AKT)/endothelial nitric oxide synthase (eNOS) signaling and promoting nitric oxide production.^4–6^ These events disrupt tight junction proteins, including occludin and claudin-5. Additional inflammatory cues, such as interleukin (IL)-9 signaling, exacerbate astrocyte activation and endothelial dysfunction.^4,6,7^ Tumor-associated inflammation increases endothelial adhesion molecules, including vascular cell adhesion molecule-1 (VCAM-1) and intercellular cell adhesion molecule-1 (ICAM-1), facilitating leukocyte adhesion and transmigration.^4,5^ These alterations create a permissive, pro-inflammatory CNS microenvironment that weakens BBB integrity.

**Figure 1.**
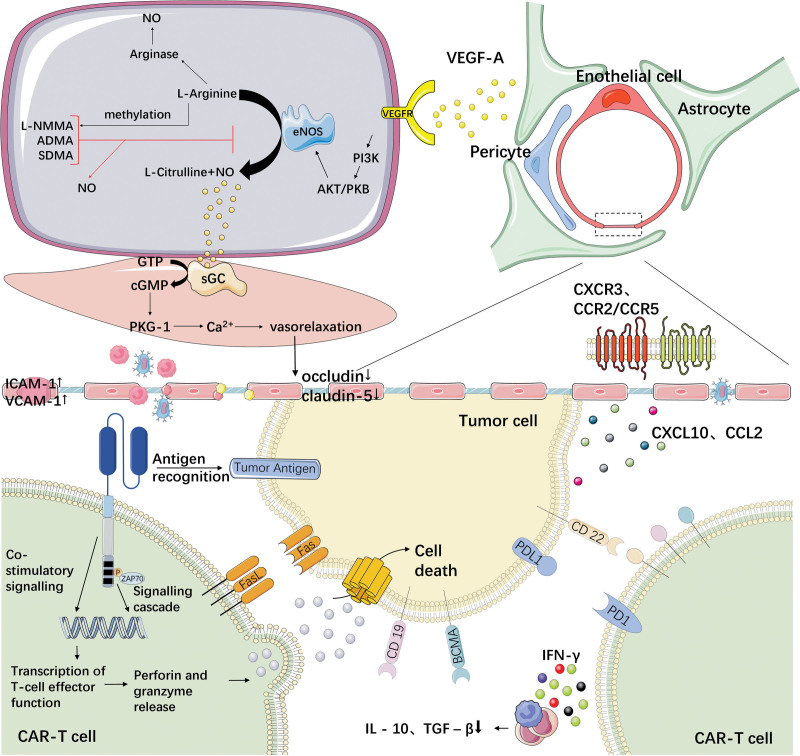
Mechanism of CAR-T therapy for CNS hematologic malignancies. Tumor cells and associated astrocytes create a permissive environment by releasing chemokines and factors that disrupt BBB integrity via eNOS/NO signaling and tight junction downregulation. CAR-T cells follow chemokine gradients into the CNS. Upon engaging tumor antigens, they exert direct cytotoxicity via perforin/granzyme B and secrete effector cytokines. CAR-T-derived cytokines amplify local immune activation and inflammatory BBB remodeling. ADMA = asymmetric dimethylarginine, AKT = protein kinase B, BBB = blood–brain barrier, CAR-T = chimeric antigen receptor T, cGMP = cyclic guanosine monophosphate, CNS = central nervous system, eNOS/NO = endothelial nitric oxide synthase/nitric oxide, GTP = guanosine triphosphate, ICAM = intercellular adhesion molecule, IFN = interferon, IL = interleukin, L-NMMA = N-monomethyl-L-arginine, PI3K = phosphatidylinositol 3-kinase, PKB = protein kinase B, PKG = cGMP-dependent protein kinase, SDMA = symmetric dimethylarginine, TGF = transforming growth factor, VCAM = vascular cell adhesion molecule, VEGF = vascular endothelial growth factor.

Following CAR-T cell entry into the CNS, the single-chain variable fragments (scFv) recognize tumor antigens, such as CD19 or BCMA, leading to intracellular signaling via ZAP-70 and PI3K.^8^ This triggers cytotoxic effector functions, including perforin and granzyme B release, resulting in direct tumor cell killing.^9^ Concurrently, CAR-T activation induces the secretion of interferon-gamma (IFN-γ), which promotes endothelial activation and inflammatory remodeling, limiting regulatory T-cell expansion and suppressive activity, while enhancing chemokine expression in tumor and stromal cells. These chemokine gradients, including CXCL10 and CCL2, facilitate recruitment of immune cells expressing CXCR3 and CCR2/CCR5, including CAR-T cells, into the CNS microenvironment (**Fig. 1**).^10–13^

Clinically, CAR-T cells have been detected in the cerebrospinal fluid (CSF) of many treated patients. Lee et al identified CAR-T cells in the CSF of 65% (11/17) of patients, whereas Lacan et al reported their presence in all 16 evaluated cases of CNS lymphoma.^14,15^ Furthermore, Hu et al^16^ demonstrated that CNS infiltration correlated with increased CSF CD3^+^ T-cell counts, higher CAR-T copy numbers, and elevated local cytokine levels in patients who developed cerebral edema.

However, CAR-T immunotherapy in the CNS represents a double-edged sword. BBB disruption associated with malignant infiltration facilitates immune cell trafficking into the CNS, thereby enhancing therapeutic access to tumor sites. Tumor-associated inflammation and chemokine gradients contribute to this permissive microenvironment that enables CAR-T cell entry. Conversely, following CNS infiltration, CAR-T–mediated immune activation amplifies local inflammatory signaling. Cytokine accumulation within the CNS may exacerbate endothelial dysfunction and BBB instability, thereby increasing the risk of neurotoxicity.^13,17^ Moreover, the CNS tumor microenvironment, characterized by immunosuppressive and metabolic constraints, can limit CAR-T persistence while sensitizing surrounding neural tissues to inflammatory injury. These processes underscore the dual therapeutic and pathological consequences of CAR-T activity within the CNS.^18^

CAR-T therapy in CNS malignancies offers substantial therapeutic potential, including potent tumor clearance and immune microenvironment reprogramming. However, these benefits are accompanied by significant risks, including CRS, BBB disruption, and neurotoxicity. Therefore, vigilant monitoring and timely management of adverse effects remain essential.

## 3. CLINICAL RESEARCH

Clinical studies of CAR-T therapy in CNS hematologic malignancies have primarily focused on lymphoma, ALL, and MM.

Efficacy and safety profiles differ across these diseases, reflecting distinct biological patterns. In CNS lymphoma, outcomes and neurotoxicity risks vary significantly between primary (PCNSL) and secondary CNS lymphoma (SCNSL), driving differential needs for consolidation and long-term disease surveillance. In CNS-ALL, CAR-T cells achieve high rates of simultaneous marrow and CNS remission, although high tumor burden may amplify the risk of neurological toxicity. Conversely, CNS-MM exhibits profound early responses to BCMA-targeted CAR-T therapy, but is challenged by delayed neurological events, including rare Parkinson-like syndromes, and suboptimal durability. These disease-specific differences suggest that CNS-directed CAR-T therapy is a spectrum of approaches, each requiring tailored strategies. The following sections review key clinical data for each category, focusing on improving efficacy, reducing neurotoxicity, and extending remission durability.

### 3.1 Lymphoma

#### 3.1.1. Primary central nervous system lymphoma

PCNSL is a rare extranodal B-cell non-Hodgkin lymphoma, representing 2% to 3% of all NHL cases.^19^ It has a poor prognosis, with a median OS of approximately 1.3 years and 3- and 5-year OS rates of 37.7% and 30.5%, respectively.^20^

Evidence on CAR-T therapy for PCNSL remains limited. A meta-analysis of 30 patients treated with CD19 CAR-T cells reported an ORR of 64% (median remission duration: 8.97 months). The CRR evolved over time; 31% at day 28, 40% at day 90, and 37% at 6 months. Adverse events were common, with CRS occurring in 70% of patients (13% with grade 3–4) and ICANS in 53% (18% with grade 3–4).^1^ A retrospective analysis by the EBMT and GoCART consortium included 16 patients treated with Axi-cel or Tisa-cel. After 1 and 2 years, progression-free survival (PFS) and OS were 47% and 62%, and 25% and 46%, respectively. Outcomes were comparable to those in SCNSL, and active CNS disease at infusion did not significantly influence prognosis. CRS and ICANS occurred in 88% and 50% of patients, respectively, with only 2 grade ≥3 events.^21^ These results suggest that anti-CD19 CAR-T therapy can achieve meaningful responses in PCNSL with manageable toxicity.

#### 3.1.2. Secondary central nervous system lymphoma

SCNSL refers to systemic lymphoma that spreads to the brain, spinal cord, eyes, or cranial structures and is commonly accompanied by extracranial disease.^22^ SCNSL occurs in 4% to 6% of patients with lymphomas.^23^ In diffuse large B-cell lymphoma (DLBCL), the median interval from diagnosis to CNS involvement is approximately 9 months, and 80% of cases arise after first-line therapy.^19^ Prognosis remains poor, with a median OS of <6 months.^24,25^

The first report of CAR-T efficacy in SCNSL was published in 2017.^26^ Thereafter, multiple small cohort studies have been conducted. Epperla et al^27^ analyzed 144 patients with SCNSL treated with CD19 CAR-T cells from the CIBMTR registry. Among 136 evaluable patients, the 100-day ORR and CRR were 68% and 53%, respectively. After 2 years, non-relapse mortality was 5%, whereas relapse/progression occurred in 74% of patients. Two-year PFS and OS were 21% and 34%, respectively. CRS occurred in 75% (grade ≥3, 12%) and ICANS in 36% (grade ≥3, 25%), with no ICANS-related deaths.^27^ This pronounced disparity between robust initial response and poor durability underscores the imperative for novel consolidation or maintenance strategies to prolong remission in SCNSL.

The risk of ICANS and CRS in CNS lymphoma is comparable to that in DLBCL without CNS involvement.^28–30^ However, in one study, the incidence of ICANS was 44.4% in PCNSL and 66.7% in SCNSL after CD19 CAR-T therapy, suggesting higher neurotoxicity in SCNSL. Neurotoxicity in SCNSL may be driven by systemic inflammation associated with high tumor burden, whereas in PCNSL, it may reflect localized CNS immune activation.^31^ SCNSL treatment commonly employs Axi-cel (containing a CD28 co-stimulatory domain, associated with greater neurotoxicity), whereas PCNSL treatment more often uses Tisa-cel.^1^ Research outcomes of CAR-T therapy in CNS lymphoma are summarized in Table 1.

**Table 1:** Efficacy of CAR-T therapy in patients with CNSL.

Authors	Target	N	Age (y)	Diagnosis	CRS	ICANS	Response	Survival
Siddiqi et al^32^	CD19	5	49 (42–53)	PCNSL	Total: 100% (5/5) G3: 0	Total: 100% (5/5) G3: 20% (1/5)	CR: 3 SD: 2	176 d (13–520)
Alcantara et al^33^	CD19	9	67 (48–75)	PCNSL	Total: 78% (7/9) G3: 11% (1/9)	Total: 56% (5/9) G3–4: 22% (2/9)	PD: 4 CR: 5	mPFS 122 d
Frigault et al^34^	CD19	12	63 (34–81)	PCNSL	Total: 58% (7/12) G3: 0	Total: 50% (6/12) G3: 8% (1/12)	CR: 6 PR: 1 PD: 5	NR
Zhou et al^35^	CD19/CD22	10	39 (18–66)	PCNSL	Total: 80% (8/10) G3: 1 (1/10)	Total: 10% (1/10) G3: 0	CR: 5 PR: 1 PD: 4	mPFS 4.72 mo 2 y PFS 30%
Choquet et al^36^	CD19	25	68 (34–76)	PCNSL	Total: 92% (23/25) G3–4: 8% (2/25)	Total: 68% (17/25) G3–4: 20% (5/25)	CR: 16 PR: 4 PD: 5	mPFS 8.4 mo mOS 21.2 mo
Frigault et al^37^	CD19	8	50 (17–79)	SCNSL	Total: 88% (7/8) G3: 0	Total: 38% (3/8) G3: 0	CR: 3 PR: 1 PD: 4	NR
Zhang et al^38^	CD19 (11) CD19/CD20 (2) CD19/CD22 (2)	15	51 (31–66)	SCNSL	Total: 73% (11/15) G3: 0	Total: 20% (3/15) G4: 7% (1/15)	CR: 9 PR: 2	mPFS 4 mo OS 9 mo
Ghafouri et al^39^	CD19	7	63 (46–76)	SCNSL	Total: 86% (6/7) G3: 0	Total: 57% (4/7) G3–4: 29% (2/7)	CR: 4	mEFS 4.4 mo mOS 8.8 mo
Strati et al^40^	CD19	8	18–85	SCNSL	Total: 0	Total: 100% (8/8) G3–4: 63% (5/8)	NR	NR
Karschnia et al^41^	CD19	10	55 (35–70)	SCNSL	Total: 100% (10/10) G3: 10% (1/10)	Total: 60% (6/10) G3–4: 30% (3/10)	CR: 2 PR: 2 SD: 3 PD: 3	MST 7 mo PFS 3 mo
Yuen et al^42^	CD19	14	58	SCNSL	Total: NR G4: 7% (1/14)	Total: 43% (6/14) G3–4: 29% (4/14)	CR: 6 PR: 1 PD: 7	NR
Nastoupil et al^43^	CD19	21	60 (21–83)	SCNSL	Total: NR	Total: NR	CR:9 PD: 12	12 mo PFS 44% OS 56%
Ayuk et al^44^	CD19	28	NR	SCNSL	Total: 86% (24/28) G3: 21% (6/28)	Total: 46% (13/28) G3–4: 14% (4/28)	CR: 9 PR: 9 PD: 7 SD: 3	mOS 21 mo mPFS 3.8 mo 12 mo PFS 41% 12 mo OS 63%
Bennani et al^45^	CD19	15	58 (25–83)	SCNSL	Total: 93% (14/15) G3: 13% (2/15)	Total: 87% (13/15) G3: 33% (5/15)	CR: 10 PD: 2	NR
Abramson et al^28^	CD19	7	54–70	SCNSL	Total: NR	Total: 29% (2/7) G3: 29% (2/7)	CR: 3 PD: 3	NR
Li et al^46^	CD19/CD22	5	39 (18–60)	1 PCNSL/4 SCNSL	Total: 100% (5/5) G3: 0	Total: 40% (2/5) G4: 20% (1/5)	PR: 3 CR: 2	OS 100% mPFS 3 mo
Liu et al^47^	CD19 (3) CD20 (4)	7	48 (34–66)	1 PCNSL/6 SCNSL	Total: 100% (7/7) G3: 0	Total: 0	CR: 4 PR: 3	Final OS rate 57.1%
Wu et al^48^	CD19/CD22	44	42 (23–65)	4 PCNSL/9 SCNSL	Total: 71% (25/35) G3: 6% (2/35)	Total: 49% (19/39) G3–4: 31% (12/39)	CR: 8 PD: 2	1 y PFS 74.6% 1 y OS 82.5%
Yu et al^49^	CD19	22	56 (29–70)	12 PCNSL/10 SCNSL	Total: 73% (16/22) G3: 5% (1/22)	Total: 36% (8/22) G3: 5% (1/22)	CR: 15 PR: 5 SD: 1 PD: 1	1 y OS 79.2% 1 y PFS 64.4%
Karschnia et al^31^	CD19	44	NR	17 PCNSL/27 SCNSL	NR	Total: 59% (26/44) G3: 16% (7/44)	CR: 18	mPFS 2.0 ± 0.7 mo
Zhang et al^50^	ssCD19	14	61 (20–70)	8 PCNSL/6 SCNSL	Total: 57% (8/14) G3: 7% (1/14)	Total: 14% (2/14) G3: 0	CR: 8 PR: 5	NR

CAR-T = chimeric antigen receptor T, CNSL = central vervous system lymphoma, CR = complete response, CRS = cytokine release syndrome, ICANS = immune effector cell–associated neurotoxicity syndrome, mEFS = median event free survival, mOS = median overall survival, mPFS = median progression-free survival, MST = median survival time, NR = not reported, OS = overall survival, PCNSL = primary central nervous system lymphoma, PD = progression of disease, PFS = progression-free survival, PR = partial response, SCNSL = secondary central nervous system lymphoma, SD = stable disease.

### 3.2 Acute lymphoblastic leukemia

CNS-ALL is an extramedullary manifestation of leukemia, resulting from leukemic cell infiltration into the meninges, neural tissues, and other CNS structures. The incidence at diagnosis in adults is <10%.^51–53^ Without prophylaxis, CNS involvement can occur in approximately 75% of patients within 1 year.^54^ Prognosis is poor, with a 5-year survival rate <30% and a median survival of 6 months.^54,55^ Allogeneic stem cell transplantation (ASCT) is an important treatment option.

The first proof-of-concept for CD19 CAR-T therapy in CNS-ALL was demonstrated by Jin et al,^2^ who reported clinical improvements in 2 patients, including complete resolution of imaging abnormalities and achievement of CSF minimal residual disease negativity. This promise has been substantiated by the largest cohort study (n = 48), which demonstrated that CAR-T therapy can achieve concurrent high-rate remissions in the bone marrow (87.5%) and sanctuary CNS site (85.4%). The study reported a median OS of 16.0 months, a significant improvement in this poor-prognosis population, despite a median PFS of 8.7 months. The 12-month cumulative relapse rate was significantly lower in the CNS (11.3%) than in the bone marrow (31.1%), underscoring the potency of CAR-T cells within the CNS compartment. Regarding safety, grade ≥3 toxicities were observed in a few patients (CRS, 18.8%; ICANS, 22.9%), and these events, including those associated with a high CNS tumor burden, were effectively managed with timely intervention.^56^

This evidence firmly establishes CD19 CAR-T therapy as a highly effective and clinically feasible strategy for achieving dual remission in R/R B-ALL with CNS involvement. The evidence is summarized in Table 2.

**Table 2:** Efficacy of CAR-T therapy in patients with R/R ALL involving the CNS.

Authors	Target	N	Age (y)	Diagnosis	CRS	ICANS	Response	Survival
Tan et al^57^	CD19	12	8 (1–13)	B-ALL	Total: 92% (11/12) G3: 17% (2/12)	Total: 83% (10/12) G3–4: 33% (4/12)	CR: 12	NR
Shalabi et al^58^	CD19	13	NR	B-ALL	Total: NR	Total: 38% (5/13) G3–4: 15% (2/13)	NR	NR
Qi et al^56^	CD19	48	31 (6–68)	B-ALL	Total: 90% (43/48) G3–4: 17% (8/48) G5: 2% (1/48)	Total: 38% (18/48) G3–4: 23% (11/48)	PR: 41	mOS 16.0 mo mEFS 8.7 mo
Leahy et al^59^	CD19	66	12 (8–17)	B-ALL	Total: 80% (53/66) G3–4: 20% (13/66)	Total: 58% (38/66) G3–4: 12% (8/66)	CR: 64	NR
Jacoby et al^60^	CD19	55	NR	B-ALL	Total: 65% (36/55) G3–4: 7% (4/55)	Total: 38% (21/55) G3–4: 9% (5/55) G5: 2% (1/55)	CR: 51	NR
Zhang et al^61^	CD19/CD22	26	NR	B-ALL	Total: 100% (26/26) G3: 0	Total: 42% (11/26) G3: 8% (2/26)	CR: 25	NR
Yang et al^62^	CD7	24	23 (4–44)	T-ALL	NR	NR	CR: 24	NR

B-ALL = B-cell acute lymphoblastic leukemia, CAR-T = chimeric antigen receptor T, CNS = central nervous system, CR = complete response, CRS = cytokine release syndrome, ICANS = immune effector cell–associated neurotoxicity syndrome, mOS = median overall survival, mPFS = median progression-free survival, NR = not reported, PR = partial response, R/R = relapsed/refractory.

### 3.3 Multiple myeloma

CNS involvement in MM is rare, occurring in approximately 1% of cases,^63,64^ and usually appears in the R/R setting. No standard therapy exists. Conventional treatments include systemic chemotherapy, local radiotherapy, intrathecal chemotherapy (dexamethasone, methotrexate, and cytarabine), and autologous stem cell transplantation.^65–67^ Outcomes are poor, with median OS <6 months.^68^

BCMA-targeted CAR-T therapy has demonstrated promising activity in CNS-MM. Wang et al^69^ demonstrated that BCMA CAR-T cells, detectable in the CSF, confirmed BBB penetration and induced deep responses in all 4 patients (CR, 3; PR, 1) with only low-grade CRS. A larger multicenter U.S. cohort (n = 10) supported this efficacy, reporting an overall response rate of 80% and a 100% CNS response rate, with ≥70% of patients achieving stringent CR or excellent PR. Neurotoxicity was generally manageable, with only 1 case of grade 3 ICANS and no grade 4 events. Delayed neurotoxicities were observed but were reversible, with no Parkinson-like syndromes observed. Importantly, patients who responded to bridging therapy experienced superior survival outcomes, underscoring the relevance of pretreatment disease control to CNS efficacy. Despite robust early responses, median PFS was limited to 6.3 months, highlighting inadequate persistence as a key barrier and supporting the need for maintenance strategies and prospective validation.^3^

A concerning delayed neurotoxicity observed in a subset of patients with CNS-MM receiving BCMA CAR-T therapy is the emergence of Parkinson-like symptoms, reported in 0% to 9% of cases across studies.^3^ Although low-level BCMA expression has been reported in certain neural tissues, including regions involved in motor control, direct evidence supporting a causal relationship between neuronal BCMA expression and these clinical manifestations remains limited. The underlying mechanisms of this delayed neurotoxicity are unclear, and further studies are required to elucidate its pathophysiology.^70–72^ Compared with other CNS hematologic malignancies, patients with CNS-MM rarely experience severe (grade ≥3) CRS or ICANS. The safety profile of BCMA CAR-T in CNS-MM is manageable in the acute phase. However, delayed neurotoxicities, including rare infectious and neurocognitive complications, were observed in 20% of patients in a real-world study, highlighting the need for long-term monitoring and post-marketing surveillance to better understand the spectrum of delayed adverse events in this population.^3^ Existing evidence is summarized in Table 3.

**Table 3:** Efficacy of BCMA-directed CAR-T therapy in patients with MM involving the CNS.

Authors	N	Age (y)	CRS	ICANS	Response	Survival
Wang et al^69^	4	57 (53–69)	Total: 100% (4/4) G3: 0	Total: 0	CR: 3 PR: 1	NR
Zhang et al^73^	1	56	Total: 100% (1/1) G3: 0	Total: 100% (1/1) G4: 100% (1/1)	PR	NR
Gaballa et al^3^	10	58	Total: 80% (8/10) G3: 0	Total: 30% (3/10) G3: 10% (1/10)	Central relief: 10	mPFS 6.3 mo mOS 13.3 mo
Maulhardt et al^74^	10	61 (47–71)	Total: 90% (9/10) G3: 20% (2/10)	Total: 20% (2/10) G3: 0	CR: 6 PR: 2	mPFS 10.5 mo mOS 12.9 mo

BCMA = B-cell maturation antigen, CAR-T = chimeric antigen receptor T, CNS = central nervous system, CR = complete response, CRS = cytokine release syndrome, ICANS = immune effector cell–associated neurotoxicity syndrome, MM = multiple myeloma, mOS = median overall survival, mPFS = median progression-free survival, NR = not reported, PR = partial response.

## 4. FUTURE RESEARCH DIRECTIONS

**Figure 2.**
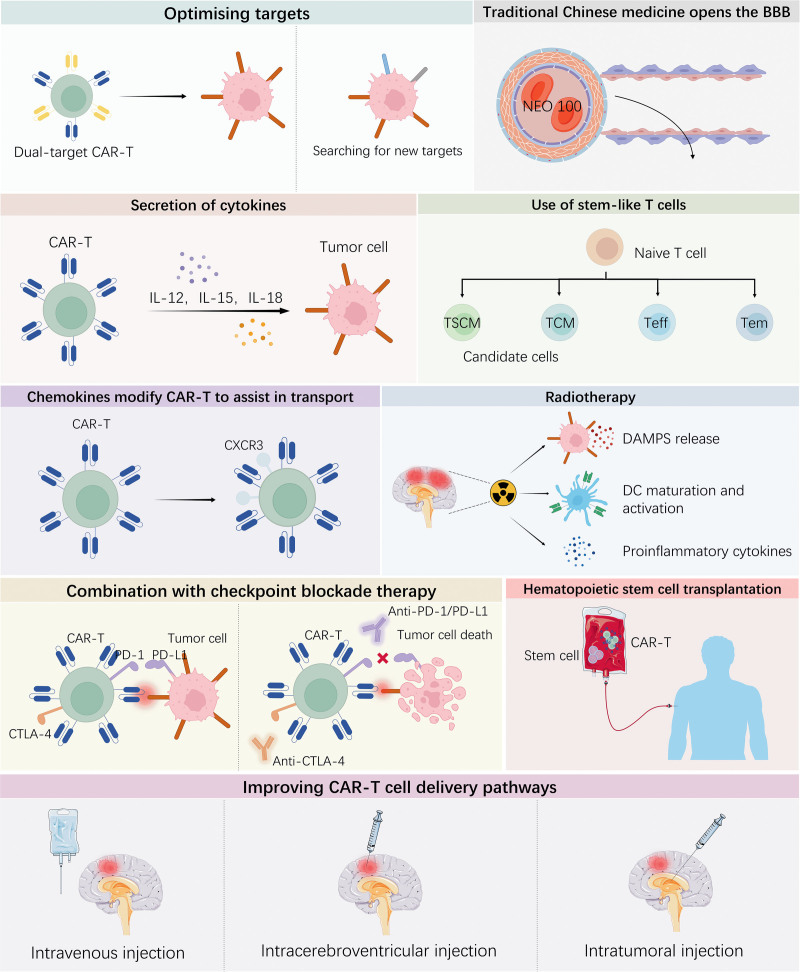
Strategies for enhancing CAR-T therapy against CNS hematologic malignancies. Approaches include (A) multi-antigen or bispecific CARs to mitigate antigen escape, (B) adjunctive agents or technologies to transiently increase BBB access, (C) engineered cytokine secretion for persistence, (D) enrichment of T memory stem cells/central memory T cells phenotypes for durability, (E) chemokine receptor modification to improve CNS homing, (F) radiotherapy as bridging or debulking, (G) checkpoint blockade to prevent exhaustion, (H) ASCT to reset immune milieu, and (I) optimized delivery routes (intravenous vs intraventricular/intratumoral) to balance efficacy and systemic toxicity. ASCT = allogeneic stem cell transplantation, BBB = blood–brain barrier, CAR-T = chimeric antigen receptor T, CNS = central nervous system, CTLA = cytotoxic T-lymphocyte–associated protein, DAMPs = damage-associated molecular patterns, DC = dendritic cells, IL= interleukin, PD-L1 = programmed death-ligand 1, TCM = central memory T cells, Teff = effector T cells, Tem = effector memory T cells, TSCM = stem cell memory T cells.

CAR-T therapy translation for CNS hematologic malignancies to routine practice is impeded by several interconnected barriers, including suboptimal CNS trafficking, antigen escape, an immunosuppressive tumor microenvironment, and off-target toxicities. Therefore, the field must optimize CAR designs, refine delivery routes, develop novel BBB modulation technologies, devise rational combination therapies, and implement robust toxicity management strategies. Here, we outline the most promising directions to redefine CNS-targeted CAR-T therapy. These strategies are illustrated in Figure 2.

### 4.1 Optimizing CAR-T cell design

#### 4.1.1 Chemokine receptor modification

The ability of CAR-T cells to migrate efficiently into the CNS is a key determinant of therapeutic efficacy. Chemokines secreted by CNS tumors and inflamed neural tissue, such as CXCL10 (IP-10), can be harnessed to guide CAR-T trafficking. Engineering CAR-T cells to express the corresponding chemokine receptors enhances their chemotaxis and accumulation at tumor sites.^11,75^ Studies in patients with SCNSL treated with CD19 CAR-T cells have demonstrated that higher IP-10 expression in CNS lesions correlates with CXCR3-mediated migration, suggesting that chemokine receptor modification may improve local infiltration while minimizing systemic distribution.^76^

#### 4.1.2 Dual-target and multispecific CARs

Antigen loss or downregulation is a key mechanism of relapse after single-antigen CAR-T therapy.

By recognizing multiple tumor antigens at once, dual-targeted or bispecific CAR-T cells can preserve their cytotoxicity, although one antigen is lost, effectively lowering the risk of immune escape.^77^ Li et al^46^ reported 5 patients with relapsed/refractory B-cell CNS lymphoma treated sequentially with anti-CD19 and anti-CD22 CAR-T cells. All 5 patients responded (CR, 40%; PR, 60%). CRS occurred in all patients, whereas 2 developed ICANS; both toxicities were effectively managed.^46^ Zou et al^78^ applied tandem CD19/CD22 dual-targeted CAR-T therapy in patients with relapsed/refractory PCNSL, followed by maintenance with additional agents after treatment. Stringent complete response (sCR) was achieved, with durability confirmed during a 35-month follow-up period.^78^ Zhou et al^79^ demonstrated that anti-BCMA/GPRC5D bispecific CAR-T cells retain cytotoxicity, although one antigen is absent, by simultaneously recognizing 2 independent targets, eradicating BCMA- or GPRC5D-negative tumor subclones, thereby significantly improving RR and reducing recurrence associated with antigen escape.^79^

#### 4.1.3 Suicide gene as a safety switch for CAR-T therapy

Switchable safety mechanisms, such as herpes simplex virus thymidine kinase (HSV-TK), enable the rapid elimination of CAR-T cells in case of severe toxicities. Following transduction, ganciclovir is phosphorylated by HSV-TK, generating cytotoxic metabolites that efficiently induce apoptosis and eliminate CAR-T cells. This strategy is relevant in CNS malignancies, in which ICANS may cause irreversible neurological damage; timely CAR-T cell ablation preserves neurological function and enhances therapeutic safety.^80^ Genetic modification may compromise CAR-T cell fitness, and BBB may hinder the delivery of prodrugs. Additionally, viral vector–mediated gene delivery may elicit immune responses against vector-encoded epitopes, compromising the persistence and efficacy of transduced CAR-T cells. Such immune recognition represents an important consideration for suicide gene–based safety strategies in CNS-directed CAR-T therapies. However, the continued optimization of suicide gene strategies is essential to improve the safety of CAR-T therapy for CNS diseases.

### 4.2 CAR-T cell delivery routes to the brain

The effectiveness of CAR-T therapy for CNS tumors depends on effective delivery of CAR-T cells to the target sites. Intravenous administration often results in limited localization and expansion of CAR-T cells within CNS tumors. Intraventricular administration delivers CAR-T cells into the CSF, facilitating distribution to periventricular areas and dissemination within the brain parenchyma. Preclinical studies in mouse models have shown that intracerebroventricular delivery of anti-CD19 CAR-T cells is more effective than intravenous administration. However, these findings were observed in immunodeficient animals, and clinical application remains limited. Alternatively, direct intratumoral injections bypass the BBB, enhancing local infiltration and tumor regression. However, the broader applicability and long-term efficacy of local delivery strategies remain lacking.^81,82^ Tumor location, disease characteristics, and therapeutic goals must be considered in selecting an appropriate delivery route. Although intracerebroventricular or intracerebral delivery enhances local CAR-T accumulation, its distribution within the CNS remains spatially restricted. Both studies indicate that CAR-T activity is confined to locally conditioned regions, with limited penetration beyond the immediate microenvironment, suggesting that local delivery does not fully overcome intrinsic anatomical and microenvironmental barriers of the CNS.

### 4.3 BBB penetration technology

#### 4.3.1 Low-intensity focused ultrasound combined with microbubbles

Low-intensity focused ultrasound combined with microbubbles (LIFU-MB) transiently increases BBB/BTB permeability by inducing MB oscillation under focused ultrasound, leading to reversible modulation of endothelial tight junctions and enhanced transcytosis. Although preclinical studies have demonstrated improved CNS drug delivery, clinical translation remains limited. Major challenges include the lack of standardized ultrasound parameters, interindividual variability related to skull properties and neuropathology, and a narrow therapeutic window lasting only several hours. Moreover, the long-term safety of repeated BBB modulation, including potential sterile neuroinflammation or microvascular injury, remains unclear, limiting broader clinical application.^83^

#### 4.3.2 Permeability enhancer

Intra-arterial infusion of hyperosmolar mannitol (20%–25%) is the most established method for transient BBB disruption. Mannitol induces reversible BBB opening through β-catenin–mediated loosening of endothelial tight junctions, with timing critically influencing delivery efficiency. However, this approach is inherently non-selective, allowing uncontrolled entry of circulating proteins and potentially neurotoxic substances into normal brain tissue. Additionally, the requirement for intra-arterial catheterization and general anesthesia, along with poor controllability of BBB opening, limits its clinical applicability. Consequently, mannitol-based BBB disruption remains restricted to selected oncologic settings.^84^

### 4.4 Combination therapy strategy

Combining CAR-T therapy with immunomodulatory drugs, chemotherapeutic agents with BBB penetration, or radiotherapy can enhance antitumor efficacy via complementary mechanisms. These combination strategies improve patient RR and survival outcomes. Additionally, rapid and individualized bridging regimens are important in stabilizing highly aggressive disease before CAR-T infusion. The principal combination strategies under investigation are summarized below. The existing combination strategies are summarized in Table 4.

**Table 4:** Clinical research on CAR-T combined therapy for CNS hematological diseases.

Authors	N	Target	Combination agent	Diagnosis	CRS	ICANS	Response	Survival
Akingbemi et al^86^	3	CD19	SRT	SCNSL	Total: 67% (2/3) G3: 0	Total: 33% (1/3) G3: 0	CR: 2 PD: 1	NR
Lionel et al^93^	4	CD19	Acalabrutinib/Zanubrutinib	CNS-MCL	Total: 75% (3/4) G3: 0	Total: 50% (2/4) G3: 50% (2/4)	CR: 3 PD: 1	NR
Jia et al^88^	1	CD19	ASCT Atezolizumab	SCNSL	Total: 100% (1/1) G3: 0	Total: 100% (1/1) G3: 0	NR	NR
Zou et al^78^	1	CD19/CD22	Camrelizumab Lenalidomide Ibrutinib	SCNSL	Total: 100% (1/1) G3: 0	Total: 0	CR	NR
Zhang et al^90^	1	BCMA	Pomalidomide	CNS-MM	Total: 100% (1/1) G3: 0	Total: 0	sCR	NR
Zhang et al^94^	1	CD19	Zanubrutinib Tislelizumab	SCNSL	Total: 0	Total: 0	CR	NR
Xue et al^87^	8	CD19	ASCT	R/R CNS B-cell lymphoma	Total: 100% (8/8) G3–4: 50% (4/8)	Total: 38% (3/8) G3–4: 25% (2/8)	CR: 7 PD: 1	NR
Ahmed et al^95^	5	CD19	WBRT	SCNSL	Total: 60% (3/5) G3: 20% (1/5)	Total: 40% (2/5) G3: 20% (1/5)	CR: 3 PD: 2	mPFS 83 d (28–219 d)
Shi et al^96^	27	CD19(24) CD20(2) CD19/CD22(1)	WBRT	19 PCNSL/8 SCNSL	Total: 48% (13/27) G3: 0	Total: 30% (8/27) G4: 4% (1/27)	CR: 23 PR: 1	1 y PFS 61.3% 1 y OS 56.6%
He et al^97^	3	CD19	Intrathecal chemotherapy	R/R CNS B-ALL	Total: 67% (2/3) G3: 0	Total: 67% (2/3) G3: 0	CR: 3	NR
Zhou et al^35^	29	CD19/CD22	ASCT	8 PCNSL/21 SCNSL	Total: 93% (27/29) G3: 7% (2/29)	Total: 21% (6/29) G3: 0	CR: 21 PR: 3 PD: 2	2 y PFS 65.52%
Yu et al^98^	9	CD19/CD22	Tislelizumab Zanubrutinib/Orelabrutinib/Ibrutinib	3 PCNSL/6 SCNSL	NR	Total: NR G3: 0	CR: 8	2 y DOR 100%

ASCT = autologous stem cell transplant, CAR-T = chimeric antigen receptor T, CNS = central nervous system, CR = complete response, CRS = cytokine release syndrome, DOR = duration of remission, ICANS = immune effector cell–associated neurotoxicity syndrome, MCL = mantle cell lymphoma, MM = multiple myeloma, mPFS = median progression-free survival, NR = not reported, OS = overall survival, PCNSL = primary CNS lymphoma, PD = progression of disease, PR = partial response, SCNSL = secondary CNS lymphoma, sCR = stringent complete response, SRT = stereotactic radiotherapy, WBRT = whole-brain radiotherapy.

#### 4.4.1 CAR-T cell therapy combined with BTK inhibitors

Preclinical studies indicate that BTK inhibitors enhance CAR-T cell expansion, reduce CD19 CAR-T exhaustion, prolong persistence in vivo, decrease immunosuppressive cell populations, and increase cytotoxicity. BTK inhibitors administered before or concurrently with CAR-T infusion may improve efficacy by lowering tumor burden and maintaining a more permissive immune microenvironment. Combination therapy reduces CAR-T exhaustion while supporting cellular proliferation and sustained function. In a small case series (n = 4), BTK inhibitors were applied as bridging therapy before brexucabtagene autoleucel (BA) and maintenance after infusion; the regimen was well-tolerated and produced encouraging responses in R/R mantle cell lymphoma (MCL) with CNS involvement.^85^ These findings suggest BTK inhibitors may represent an ideal combination strategy for CNS-MCL.

#### 4.4.2 CAR-T cell therapy combined with PD-1/PD-L1 inhibitors

Immune checkpoint blockade can promote CAR-T proliferation, extend persistence, and enhance antitumor efficacy. Preclinical and clinical evidence suggest PD-1 inhibitors have potential in PCNSL treatment by modulating the CNS-suppressive tumor microenvironment. Zou et al^78^ reported that several patients who had failed to respond to BTK and PD-1 inhibitors before CAR-T infusion achieved clinical responses as these agents were reintroduced as maintenance therapy after CAR-T, with no notable adverse events observed during 1 year of follow-up. These findings suggest that sequential administration of dual-target CD19/CD22 CAR-T cells, followed by maintenance PD-1 and BTK inhibition, could represent a safe and effective therapeutic option for PCNSL; however, validation in larger clinical cohorts and real-world settings is warranted.

#### 4.4.3 CAR-T cell therapy combined with radiotherapy

Radiotherapy helps reduce tumor burden and acts synergistically with CAR-T therapy, enhancing overall antitumor efficacy. Bridging radiotherapy before CAR-T therapy has been employed to control systemic lymphoma and active CNS disease. Although whole-brain radiotherapy (WBRT) is commonly used for consolidation or remission, it carries significant neurotoxicity. Stereotactic radiotherapy (SRT) offers a more localized approach that markedly reduces neurocognitive toxicity; however, it has not yet become a standard practice. Akingbemi et al^86^ reported 3 patients who received SRT as bridging therapy before CAR-T treatment; all achieved durable CNS responses lasting approximately 24 months, with no residual neurocognitive deficits or severe ICANS. These results highlight the potential of SRT as an effective bridging strategy for CNS lymphoma.

#### 4.4.4 CAR-T cell therapy combined with hematopoietic stem cell transplantation

ASCT followed by CAR-T therapy leverages immune reconstitution to promote CAR-T proliferation and survival while preserving sustained immune surveillance. Xue et al^87^ reported that in 17 relapsed/refractory CNS B-cell lymphoma patients, the CRR was 100% among 8 patients receiving ASCT plus CAR-T, compared with 44.4% among 9 patients treated with CAR-T alone. The combined therapy group demonstrated prolonged PFS and OS.^87^ Similarly, Jia et al applied ASCT followed by CAR-T infusion after high-dose methotrexate chemotherapy, achieving durable CR of intracranial lesions at day 100 and 450 post-treatment, as confirmed by magnetic resonance imaging (MRI) and positron emission tomography–computed tomography (PET-CT) imaging.^88^

#### 4.4.5 CAR-T cell therapy combined with immunomodulators

Immunomodulatory drugs, such as lenalidomide and pomalidomide, exhibit CNS penetration and can modulate the tumor immune microenvironment. Zhang et al^89^ reported a patient with B-ALL and isolated CNS relapse refractory to conventional chemotherapy who achieved CR following lenalidomide administration after CAR-T therapy and hematopoietic stem cell transplantation (HSCT). In CNS-MM, pomalidomide demonstrated superior CNS penetration and efficacy if used as a bridging therapy before BCMA CAR-T treatment, reducing tumor burden without inducing neurotoxicity. Although single cases cannot establish superiority over CAR-T alone, these outcomes strongly support the investigation of combination strategies.^90,91^

#### 4.4.6 CAR-T cell therapy combined with traditional Chinese medicine formulations

Preclinical studies demonstrate that intra-arterial administration of NEO100, a high-purity perillyl alcohol, safely and reversibly increases BBB permeability by disrupting tight junctions. In a mouse model of CNS lymphoma, this approach enabled intravenously delivered CD19 CAR-T cells to achieve approximately 100% survival by facilitating their entry and eradicating intracranial tumors, without significant off-target accumulation.^92^ However, current evidence for NEO100-based BBB modulation remains limited to preclinical studies, and its safety, feasibility, and therapeutic benefit in humans remain lacking. Further clinical investigation is required before this strategy can be broadly translated into clinical practice.

## 5. CONCLUSION

CAR-T cell therapy is a pivotal treatment for CNS hematologic malignancies. Clinical evidence demonstrates that CAR-T cells can traverse the BBB and elicit robust, often rapid, antitumor responses across CNS lymphoma, leukemia, and myeloma. However, durable responses remain constrained by CNS-specific challenges, including restricted T-cell trafficking, antigen escape, a profoundly immunosuppressive microenvironment, and CNS-specific treatment-related neurotoxicities.

Therefore, the field is advancing toward the rational integration of multifaceted strategies. This includes engineering “smarter” cells with enhanced CNS homing via chemokine receptors, multi-antigen targeting, and integrated safety switches, leveraging advanced delivery approaches, such as localized administration and transient BBB modulation, and deploying rational combinations with BBB-penetrating small molecules, immunomodulators, or radiotherapy to reshape the tumor microenvironment and maximize synergistic efficacy.

Translating these next-generation CAR-T strategies from preclinical models and early-phase clinical studies into routine practice constitutes the future critical frontier. Success will depend on technological innovation and rigorous evaluation of safety and long-term efficacy via well-designed, multicenter clinical trials and real-world evidence. By systematically addressing these translational challenges, CAR-T therapy may evolve from a salvage option into a definitive, potentially curative intervention for CNS hematologic malignancies.

## ACKNOWLEDGMENTS

This review article was funded by the Fundamental Research Program of Shanxi Province (no. 202303021211224, W.T.).

The authors thank their colleagues for constructive comments and technical support.

## AUTHOR CONTRIBUTIONS

X.L., H.D., Y.Y., and J.Z. contributed to investigation, data curation, and writing-original draft. J.W. and W.T. contributed to writing-review & editing, supervision. All authors have read and approved the final manuscript.
